# Calcium-Activated-Calcineurin Reduces the *In Vitro* and *In Vivo* Sensitivity of Fluconazole to *Candida albicans via* Rta2p

**DOI:** 10.1371/journal.pone.0048369

**Published:** 2012-10-30

**Authors:** Yu Jia, Ren-Jie Tang, Lin Wang, Xiang Zhang, Ying Wang, Xin-Ming Jia, Yuan-Ying Jiang

**Affiliations:** 1 School of Pharmacy, Second Military Medical University, Shanghai, China; 2 Department of Immunology, Tongji University School of Medicine, Shanghai, China; 3 Department of Neurosurgery, Tenth People's Hospital, Tongji University School of Medicine, Shanghai, China; 4 Department of Dermatology, Changhai Hospital, Second Military Medical University, Shanghai, China; Universidade de Sao Paulo, Brazil

## Abstract

Due to the emergence of drug-resistance, first-line therapy with fluconazole (FLC) increasingly resulted in clinical failure for the treatment of candidemia. Our previous studies found that *in vitro RTA2* was involved in the calcineurin-mediated resistance to FLC in *C. albicans*. In this study, we found that calcium-activated-calcineurin significantly reduced the *in vitro* sensitivity of *C. albicans* to FLC by blocking the impairment of FLC to the plasma membrane *via* Rta2p. Furthermore, we found that *RTA2* itself was not involved in *C. albicans* virulence, but the disruption of *RTA2* dramatically increased the therapeutic efficacy of FLC in a murine model of systemic candidiasis. Conversely, both re-introduction of one *RTA2* allele and ectopic expression of *RTA2* significantly reduced FLC efficacy in a mammalian host. Finally, we found that calcium-activated-calcineurin, through its target Rta2p, dramatically reduced the efficacy of FLC against candidemia. Given the critical roles of Rta2p in controlling the efficacy of FLC, Rta2p can be a potential drug target for antifungal therapies.

## Introduction

*Candida* species are serious human fungal pathogens among immuno-compromised individuals such as patients with AIDS and patients undergoing organ transplantation, parenteral nutrition and radiation treatment for cancer [1]. *Candida* blood stream infections (candidemia) are life threatening among hospitalized immune-compromised patients, including neonates [2], [3]. Despite available therapy options, the mortality rate directly attributable to candidemia is high, ranging from 32% to 54%, depending on the population and the species studied [4], [5], [6]. Today, candidemia leads to additional ICU length of stay and costs, the annual treatment cost is at least £16.2 million [7], [8]. Although infections with non-albicans *Candida* species have emerged in recent years, *Candida albicans* is still responsible for the majority of cases [9], [10].

To date, only four classes of antifungal drugs are clinically available for treatment of systemic fungal infections: polyenes (amphotericin B), pyrimidines (particularly 5-fluorocytosine), azoles (e.g. fluconazole, FLC) and echinocandins (e.g. caspofungin) [11], [12]. Among these, azole antifungal drugs, such as FLC, are commonly used in the treatment of invasive candidiasis, and the mechanism of action of these drugs involves the blockade of ergosterol synthesis through the competitive inhibition of 14α-demethylase, which is encoded by *ERG11* gene. Consequently, the inhibition of the enzyme leads to accumulation of 14-methylated sterols that can cause disruption of fungal membranes [13]. Although these drugs have advanced the management of fungal infections, the rate of therapeutic failure remains high. Most importantly, the small number of treatment options available has resulted in wide-spread drug resistance in pathogenic species. For each of the four classes of antifungals drugs (polyenes, pyrimidines, azoles, echinocandins), drug-resistant clinical strains has been reported [14]; Azole-resistant *Candida* spp. is now particularly common among isolates from HIV-positive patients [15]. Therefore, the need of developing new antifungal strategies is pressing.

Inhibition of calcineurin-mediated azole resistance has been proposed as a novel therapeutic approach [16]. Although the precise mechanisms involved remain elusive, extensive experiments have further confirmed the benefits of such combinatorial approaches. For instance, inhibitors of calcineurin (CsA, FK506) or inhibitors of calcineurin adaptor protein HSP90 (geldanamycin, radicicol) can chemosensitize *C. albicans* cells to azoles [17], [18], [19]. Although these examples sufficiently demonstrate the potential for combination antifungal therapy, suppression of the human immune system by CsA/FK506 currently precludes the use of such inhibitors in clinics [20], [21]. While a non-immunosuppressive FK506 analogue (L-685, 818) has been identified, proprietary restrictions have currently prevented further testing [20]. Given the importance of serious mycoses in clinical medicine and the need of alternative therapies for use in patients with fungal infections, research designed to identify new antifungal targets remains a priority.

We previously established that calcium-upregulated expression of *RTA2* dramatically reduced the sensitivity of *C. albicans* to FLC by blocking its impairment to the fungal plasma membrane [22]. The expression of *RTA2* was under the control of the calcium-activated-calcineurin *via* its transcriptional factor Crz1p in *C. albicans* [22]. However, it is unclear whether calcium-activated-calcineurin mediates *in vitro* and *in vivo* responses to FLC *via* Rta2p in *C. albicans*. Moreover, the therapeutic potential of targeting Rta2p remains elusive. Here, we investigated the possible influences of *RTA2* expression on the *in vitro* and *in vivo* responses to FLC mediated by the calcium-activated-calcineurin in *C. albicans*.

## Results

### Calcium-activated-calcineurin Dramatically Reduces *in vitro* Sensitivity to FLC *via* Rta2p in *C. albicans*

It has been well documented that the phosphatase activity of calcineurin, consisted of a catalytic subunit A (encoded by *CNA*) and a regulatory subunit B (encoded by *CNB1*), can be activated by calcium ions and then regulate its target gene expression *via* transcriptional factor Crz1p [23]. In order to identify whether calcium-activated-calcineurin mediates the *in vitro* response to FLC *via* Rta2p in *C. albicans*, we sequentially disrupted two alleles of *RTA2* gene in either *cna*Δ/Δ or *crz1*Δ/Δ null mutant strains (Table 1), which were verified by Southern blot analysis with a probe specific to the *RTA2* promoter (Figure S1). And then *RTA2*, *CNA* or *CRZ1* was selectively re-introduced into either *cna*Δ/Δ *rta2*Δ/Δ or *crz1*Δ/Δ *rta2*Δ/Δ mutant at the *ade2* locus as determined by PCR (Figure S2A and S2B). Calcium treatment significantly up-regulated the expression of *UTR2*, a known target gene of the calcineurin pathway [23] in both *rta2*Δ/Δ *cna*Δ/Δ::*CNA* and *rta2*Δ/Δ *crz1*Δ/Δ::*CRZ1* complemented strains (Figure S3A), which confirmed that the function of the calcineurin pathway had been restored. The expression of *RTA2* had no changes in both *cna*Δ/Δ *rta2*Δ/Δ::*RTA2* and *crz1*Δ/Δ *rta2*Δ/Δ::*RTA2* complemented strains when treated with calcium (Figure S3B). These results confirmed that the expression of *RTA2* was under the control of the calcium-activated-calcineurin *via* its transcriptional factor Crz1p in *C. albicans*.

**Table 1:** *C. albicans* strains used in this study.

Strain	Parental strain	Genotype	Reference
RM1000	RM100	*ura3Δ::imm^434^/ura3Δ::imm^434^, his1Δ::HisG/his1Δ::HisG, iro1Δ::imm^434^/iro1Δ::imm^434^*	[55]
RM1000U	RM1000	*RM1000* * *ADE2/ade2::URA3*	This study
JXM101	JXM100	*RM1000* * *rta2Δ::hisG/rta2Δ::hisG*	[22]
JXM201	JXM101	*RM1000* * *rta2Δ::hisG/rta2Δ::hisG ADE2/ade2::RTA2/URA3*	[37]
JXM101U	JXM101	*RM1000* * *rta2Δ::hisG/rta2Δ::hisG ADE2/ade2::URA3*	This study
CAF2-1	SC5314	*ura3Δ::imm434/URA3*	[56]
DSY2091	CAF4-2	*cnaΔ::hisG/cnaΔ::hisG::URA3::hisG*	[23]
DSY2115	DSY2101	*cnaΔ::hisG/cnaΔ::hisG; LEU2::CNA::URA3*	[18]
DSY2195	DSY2188	*crz1Δ::hisG/crz1Δ::hisG::URA3::hisG*	[23]
MKY268	MKY59	*crz1Δ::hisG/crz1Δ::hisG LEU2::CRZ1/URA3*	[23]
DSY2101	DSY2091	*cnaΔ::hisG/cnaΔ::hisG*	[18]
DJY201	DSY2101	*rta2Δ::hisG/rta2Δ::hisG cnaΔ::hisG/cnaΔ::hisG*	This study
DJYCNA	DJY201	*rta2Δ::hisG/rta2Δ::hisG cnaΔ::hisG/cnaΔ::hisG ADE2/ade2::CNA/URA3*	This study
DJYRTA2	DJY201	*cnaΔ::hisG/cnaΔ::hisG rta2Δ::hisG/rta2Δ::hisG ADE2/ade2::RTA2/URA3*	This study
MKY59	DSY2195	*crz1Δ::hisG/crz1Δ::hisG*	[23]
MJY201	MKY59	*rta2Δ::hisG/rta2Δ::hisG crz1Δ::hisG/crz1Δ::hisG*	This study
MJYCRZ1	MJY201	*rta2Δ::hisG/rta2Δ::hisG crz1Δ::hisG/crz1Δ::hisG ADE2/ade2::CRZ1/URA3*	This study
MJYRTA2	MJY201	*crz1Δ::hisG/crz1Δ::hisG rta2Δ::hisG/rta2Δ::hisG ADE2/ade2::RTA2/URA3*	This study
DSY9u	DSY1024	*cdr1Δ::hisG/cdr1Δ::hisG cdr2Δ::hisG/cdr2Δ::hisG camdr1Δ::hisG/camdr1Δ::hisG flu1Δ::hisG/flu1Δ::hisG*	This study
DSJ101	DSJ100	*DSY9u* # *rta2Δ::hisG/rta2Δ::hisG*	[57]
DSJRTA2	DSJ101	*DSY9u* # *rta2Δ::hisG/rta2Δ::hisG ADE2/ade2::pCDR2-RTA2/URA3*	This study
DSJ101U	DSJ101	*DSY9u* # *rta2Δ::hisG/rta2Δ::hisG ADE2/ade2::URA3*	This study

* RM1000 background;

# DSY9u background.

And then the influences of calcium signaling on the sensitivities of above-constructed mutants to FLC were examined. The addition of calcium (1 mM) significantly reduced the sensitivities of FLC to both *cna*Δ/Δ::*CNA* and *crz1*Δ/Δ::*CRZ1* complemented strains, with an increase of MIC_80_s(Minimum Inhibitory Concentration for 80%)from 0.5 to 16 µg/ml (Table 2). However, re-introduction of anyone of *CNA*, *CRZ1* and *RTA2* could not restore the effect of calcium on reducing FLC sensitivities to complemented strains including *rta2*Δ/Δ *cna*Δ/Δ::*CNA*, *cna*Δ/Δ *rta2*Δ/Δ::*RTA2*, *rta2*Δ/Δ *crz1*Δ/Δ::*CRZ1* and *crz1*Δ/Δ *rta2*Δ/Δ::*RTA2* (Table 2). But the effect of calcium on reducing FLC sensitivity was completely restored in the *rta2*Δ/Δ::*RTA2* complemented strain, with an increase of MIC_80_s from 0.5 to 64 µg/ml (Table 2). In contrast to the results presented above, the addition of calcium (1 mM) had no effect on the antifungal activity of caspofungin in all tested strains (Table 2). Taken together, these results suggested that calcium-activated-calcineurin dramatically reduced the *in vitro* sensitivity to FLC *via* Rta2p in *C. albicans* (Figure S4).

**Table 2:** Effects of calcium on the antifungal activity *versus* mutant *C. albicans* strains.

Antifungal MIC_80_ (µg/ml)^a^		Strains						
		*cnaΔ/Δ*::*CNA*	*rta2Δ/Δ cnaΔ/Δ*::*CNA*	*cnaΔ/Δ rta2Δ/Δ*::*RTA2*	*crz1Δ/* *Δ*::*CRZ1*	*rta2Δ/Δ crz1Δ/* *Δ*::*CRZ1*	*crz1Δ/Δ rta2Δ/* *Δ*::*RTA2*	*rta2Δ/Δ*::*RTA2*
Fluconazole	–	0.5	0.125	0.06	0.5	0.125	0.125	0.5
	+1mM CaCl_2_	16	0.25	0.125	16	0.25	0.25	64
Caspofungin	–	0.3	0.6	0.6	0.3	0.3	0.15	0.3
	+1mM CaCl_2_	0.3	0.6	0.6	0.6	0.6	0.3	0.3

a MIC_80_s were determined after 48 h incubation and all experiments were performed in triplicate.

### Calcium-activated-calcineurin Blocks the Impairment of FLC to the Plasma Membrane of ***C. albicans*** *via* Rta2p

**Figure 1:**
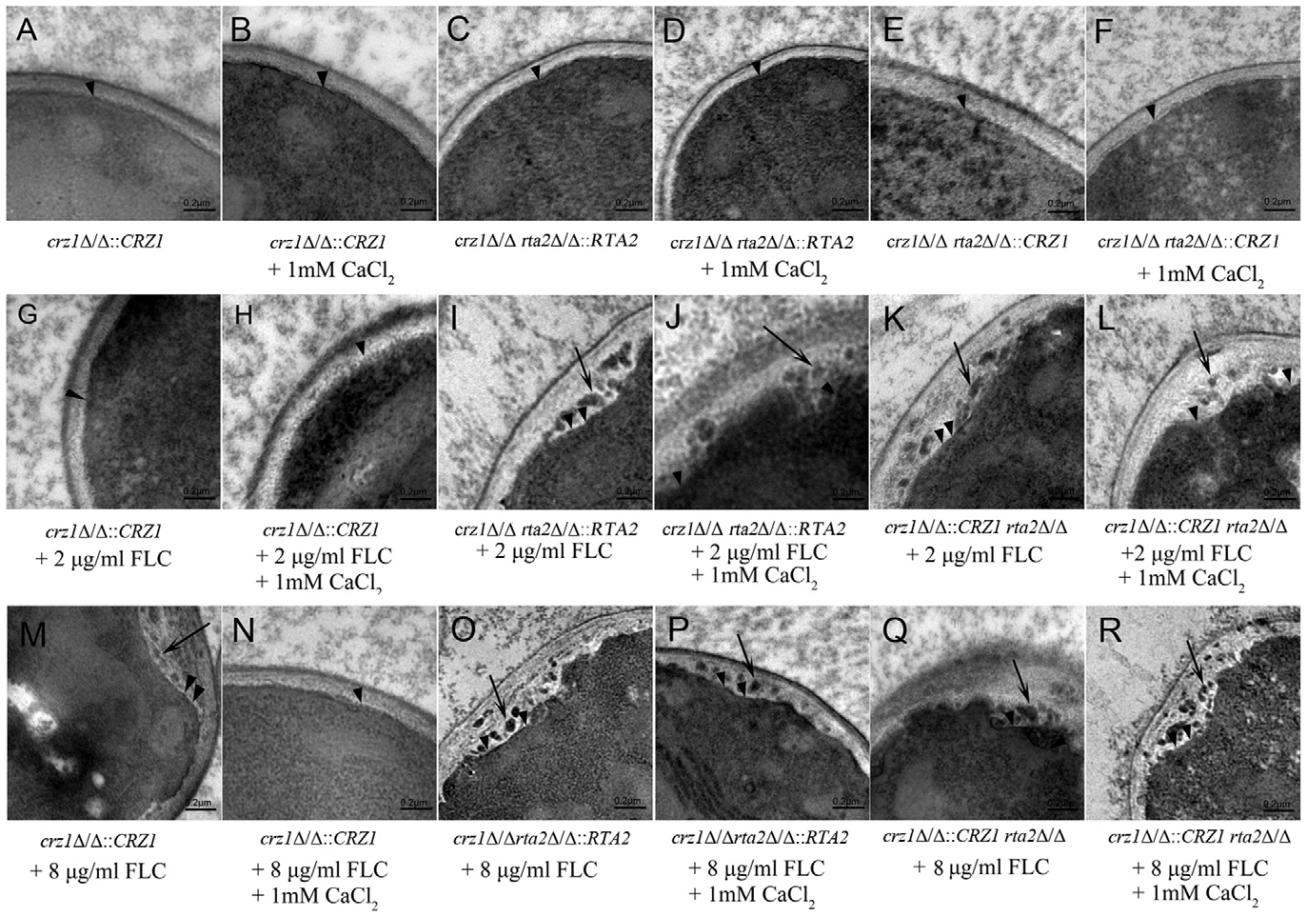
Ultra-structure of *C. albicans* cells. (**A to R**) Ultra-structural images of *crz1*Δ/Δ::*CRZ1*, *rta2*Δ/Δ *crz1*Δ/Δ::*CRZ1* and *crz1*Δ/Δ *rta2*Δ/Δ::*RTA2* complemented *C. albicans* strains in the presence or absence of agents at indicated concentrations shown on the lower. Arrow heads indicated the cytoplasmic membrane and arrows indicated the extensive solubilization of the cytoplasmic membrane. The bar represents a length of 0.2 µm.

In our previous study, it was found that the depletion of *RTA2* made the plasma membranes of *C. albicans* liable to be destroyed by FLC and calcium-upregulated expression of *RTA2* attenuated the destroying effects [22]. In the present study, transmission electron microscopy was performed to observe the ultra-structure changes of *crz1*Δ/Δ::*CRZ1*, *rta2*Δ/Δ *crz1*Δ/Δ::*CRZ1* and *crz1*Δ/Δ *rta2*Δ/Δ::*RTA2* complemented strains in the presence or absence of FLC or FLC plus calcium. High magnification of these three untreated or calcium-treated complemented strains showed intact cell membranes (Figure 1 A to F). As shown in Figure 1G and 1M, the cell membranes of the *crz1*Δ/Δ::*CRZ1* complemented strain remained intact when treated with 2 µg/ml FLC and were modestly damaged when treated by 8 µg/ml FLC. Surprisingly, the addition of calcium could completely attenuate the destroying effects of FLC to the plasma membrane of *crz1*Δ/Δ::*CRZ1* complemented strain (Figure 1N). However, re-introduction of one *CRZ1* or *RTA2* allele into *crz1*Δ/Δ *rta2*Δ/Δ mutant failed to attenuate the destroying effects of 2 µg/ml FLC to the plasma membranes of either *rta2*Δ/Δ *crz1*Δ/Δ::*CRZ1* or *crz1*Δ/Δ *rta2*Δ/Δ::*RTA2* complemented strains (Figure 1I and 1K). And extensive solubilization of intracytoplasmic inclusion bodies was seen in either *rta2*Δ/Δ *crz1*Δ/Δ::*CRZ1* or *crz1*Δ/Δ *rta2*Δ/Δ::*RTA2* complemented strains when treated by 8 µg/ml FLC (Figure 1O and 1Q). The addition of calcium also failed to attenuate the destroying effects of FLC to the plasma membranes of either *rta2*Δ/Δ *crz1*Δ/Δ::*CRZ1* or *crz1*Δ/Δ *rta2*Δ/Δ::*RTA2* complemented strains (Figure 1 P and R). Taken together, these results suggested a model in which the calcium-activated-calcineurin blocked the impairment of FLC to the plasma membrane of *C. albicans via* Rta2p.

### Rta2p is Critical for the Therapeutic Efficacy of FLC Against Candidemia

**Figure 2:**
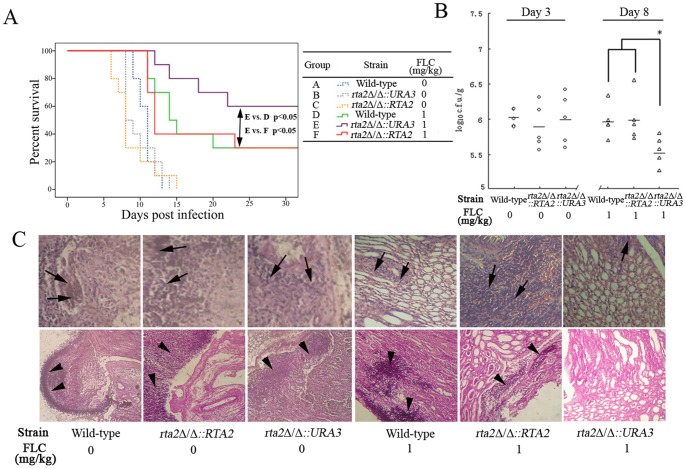
Rta2p played an important role in FLC efficacy against systemic candidiasis. (**A**) Survival curves of different groups of mice infected by *C. albicans* strains. If indicated, groups of mice received intraperitoneal FLC therapy at 1 mg/kg or placebo once a day for a week. (**B**) Kidney c.f.u. assay in mice with systemic candidiasis. Paired kidneys of untreated and FLC-treated mice were removed aseptically for c.f.u. assay on day 3 and 8 post infection, respectively. ^*^ *P*<0.05 compared with groups of mice infected with either wild-type or *rta2*Δ/Δ::*RTA2* complemented strains after receiving FLC therapy. (**C**) Histopathological analysis of kidneys from mice with systemic candidiasis. Paired kidneys of untreated and FLC-treated mice were removed aseptically for histopathological analysis on day 3 and 8 post infection, respectively. The top panels show H&E sections; the bottom panels show PAS staining in kidney sections. Arrows indicate abscesses; arrow heads indicate fungi.

It has been well documented that calcineurin is essential for the virulence of *C. albicans* in systemic infection models and for the emergence of FLC resistance to *C. albicans* [24], [25]. As a target gene of the calcineurin pathway [22], the influence of *RTA2* on either virulence or efficacy of FLC remains unknown. To examine the influence of *RTA2* on *C. albicans* virulence, groups of 20 mice were intravenously injected with the wild type, *rta2*Δ/Δ mutant and *rta2*Δ/Δ::*RTA2* complemented strains, and the survival of mice was monitored for 30 days. As shown in Figure 2A, the comparison of the survival times among groups of mice infected by the wild-type, *rta2*Δ/Δ mutant and *rta2*Δ/Δ::*RTA2* complemented strains showed no significant differences. Furthermore, both the tissue burdens and histological analysis determined at day 3 post-inoculation consistently showed similar phenotypes (Figure 2 B and 2C). These findings proved that *RTA2* was not involved in the virulence of *C. albicans*.

We then investigated the influences of *RTA2* on the efficacy of FLC against *C. albicans* in a murine model of systemic candidiasis. FLC treatment resulted in about 30% survival rate of mice infected with wild-type strain in 30 days (Figure 2A). In contrast, the disruption of *RTA2* significantly improved the survival of mice infected with *rta2*Δ/Δ mutant to 60% (Figure 2A). More importantly, this improved survival dropped to 30% when infected with *rta2*Δ/Δ::*RTA2* complemented strain (Figure 2A). When the fungal burden in renal tissues was counted at day 8 post-inoculation, we noticed that, due to FLC treatment, mice infected with *rta2*Δ/Δ mutant exhibited a lower fungal load than mice infected with either wild-type or *rta2*Δ/Δ::*RTA2* complemented strains (*P*<0.05; Figure 2B), which was consistent with the results of survival analysis. A similar trend was also shown in kidney pathological changes analysis (Figure 2C). Therefore, these results suggested that Rta2p plays a critical role on the efficacy of FLC against systemic candidiasis.

### Ectopic Expression of *RTA2* Reduced the Efficacy of FLC Against Candidemia

In order to verify the direct influences of *RTA2* on the efficacy of FLC, the expression of *RTA2* ORF was put under the control of the oestradiol-inducible *CDR2* promoter (pCDR2) and the plasmid pCDR2-RTA2 was introduced into a *C. albicans* mutant (DSJ101) with deletion of drug-resistance-related genes including *CDR1*, *CDR2*, *CaMDR1*, *FLU1* and *RTA2* at the *ade2* locus as determined by PCR (Figure S2C). Up-regulation of *RTA2* in DSJRTA2 harboring the plasmid pCDR2-RTA2 by oestradiol was confirmed by quantitative RT-PCR (Figure S3C).

**Figure 3:**
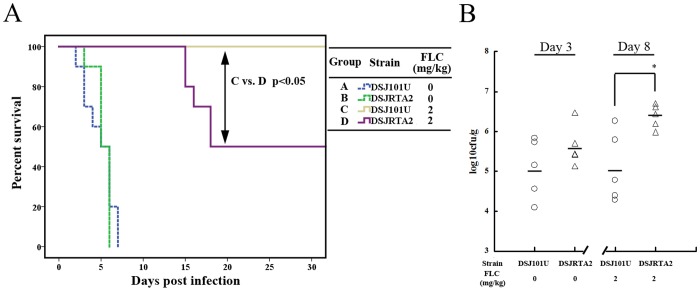
Ectopic expression of *RTA2 in vivo* significantly reduced FLC efficacy against candidiasis. (**A**) Survival curves of different groups of mice infected by DSJ101U with deletion of drug-resistance-related genes (*CDR1*, *CDR2*, *CaMDR1*, *FLU1* and *RTA2*) or DSJRTA2 carrying the plasmid pCDR2-RTA2. If indicated, groups of mice received intraperitoneal FLC therapy at 2 mg/kg or placebo once a day for a week. (**B**) Kidney c.f.u. assay in mice with systemic candidiasis. Paired kidneys of untreated and FLC-treated mice were removed aseptically for c.f.u. assay on day 3 and 8 post infection, respectively. ^*^ *P*<0.05 compared with groups of mice infected by DSJ101U with FLC therapy.

Survival analysis showed that DSJRTA2 and its parental strain (DSJ101U) had similar virulence, and all mice died within 10 days (Figure 3A). The kidney fungal burdens, determined at day 3 post-inoculation, consistently reflected virulence phenotypes observed between DSJRTA2 and DSJ101U (Figure 3B), which confirmed that *RTA2* was not involved in *C. albicans* virulence. Of note, FLC treatment made all DSJ101U-infected mice survive during the experiments (Figure 3A). However, only 50% of the DSJRTA2-infected mice survived after treatment with FLC (Figure 3A). Kidneys of mice infected with DSJRTA2 exhibited a significantly higher fungal burden than mice infected with its parental strain DSJ101U (*P*<0.05, Figure 3B). Therefore, these results suggested that Rta2p directly affects the efficacy of FLC against candidiasis.

### Calcium-activated-calcineurin, through its Target Rta2p, Significantly Reduces the Efficacy of FLC Against Candidemia

The expression of *RTA2* has been reported to be under the control of the calcium-activated-calcineurin *via* its transcriptional factor Crz1p in *C. albicans* [22], [23]. Since mammalian contains a range of ionized calcium in whole blood (0.94–1.33 mM) [26], it was examined whether the calcineurin pathway of *C. albicans* could be activated by ionized calcium in serum and then the calcium-activated-calcineurin mediated *in vivo* responses of *C. albicans* to FLC *via* Rta2p.

**Figure 4:**
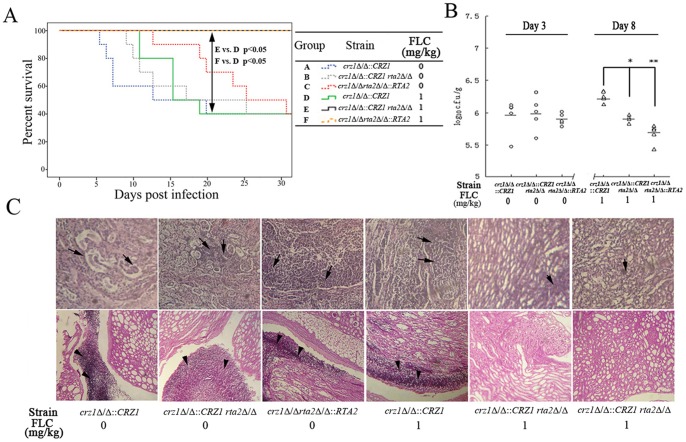
Calcium-activated-calcineurin mediated the *in vivo* response to FLC *via* Rta2p in *C. albicans*. (**A**) Survival curves of among groups of mice infected by *crz1*Δ/Δ::*CRZ1*, *rta2*Δ/Δ*crz1*Δ/Δ ::*CRZ1* or *crz1*Δ/Δ *rta2*Δ/Δ::*RTA2* complemented strains. If indicated, groups of mice received received intraperitoneal FLC therapy at 1 mg/kg or placebo once a day for a week. (**B**) Kidney c.f.u. assay in mice with systemic candidiasis. Paired kidneys of untreated and FLC-treated mice were removed aseptically for c.f.u. assay on day 3 and 8 after the infection, respectively. ^*^ *P*<0.05 or ^*^ *P*<0.01 compared with groups of mice infected by *crz1*Δ/Δ::*CRZ1* after receiving FLC therapy. (**C**) Histopathological analysis of kidneys from mice with systemic candidiasis. Paired kidneys of untreated and FLC-treated mice were removed aseptically for histopathological analysis on day 3 and 8 post infection, respectively. The top panels show H&E sections; the bottom panels show PAS staining in kidney sections. Arrows indicate abscesses; arrowheads indicate fungi.

After receiving FLC treatment, survival analysis showed that re-introduction of either one *CRZ1* or *RTA2* allele into *crz1*Δ/Δ *rta2*Δ/Δ mutant led to 100% of survival (Figure 4A). However, re-introduction of one *CRZ1* allele into *crz1*Δ/Δ mutant significantly reduced survival rate to 50% (Figure 4A). The fungal burdens of mice infected with the *crz1*Δ/Δ::*CRZ1* complemented strain were significantly higher than those infected with either the *rta2*Δ/Δ *crz1*Δ/Δ::*CRZ1* or *crz1*Δ/Δ *rta2*Δ/Δ::*RTA2* complemented strains (*P*<0.05 and *P*<0.01, respectively; Figure 4B). Histological analysis with HE staining also showed that kidney damage was aggravated in mice infected with *crz1*Δ/Δ::*CRZ1* complemented strain infected when compared with either *rta2*Δ/Δ *crz1*Δ/Δ::*CRZ1* or *crz1*Δ/Δ *rta2*Δ/Δ::*RTA2* complemented strains (Figure 4C). PAS staining also revealed a large number of fungi in kidneys from the *crz1*Δ/Δ::*CRZ1* complemented strain infected mice and hardly any fungi in mice infected with either the *rta2*Δ/Δ *crz1*Δ/Δ::*CRZ1* or *crz1*Δ/Δ *rta2*Δ/Δ::*RTA2* complemented strains (Figure 4C). Therefore, the results of H&E and PAS staining were consistent with those of survival analysis and kidney fungal burdens. Overall, ionized calcium in mice serum could not reduce the efficacy of FLC against bloodstream infection with *C. albicans* mutants due to their lack of either *CRZ1* or *RTA2*, which indicated that calcium-activated-calcineurin affects the efficacy of FLC against candidemia *via* Rta2p.

## Discussion

FLC has long been the drug of choice for candidemia, owing to its excellent safety profile and efficacy against key causative pathogens. However, because of the associated rise in FLC-resistant *Candida* infections and the increased risk of death due to FLC failure as first-line therapy [9], [27], the need of identifying new antifungal strategies to enhance FLC efficacy against *Candida* infections is pressing. A major limitation of FLC is its emergence of tolerance. As a result, many individuals require long-term treatment of FLC, and this in turn frequently results in the selection of FLC-resistant fungal strains. In *C. albicans*, calcineurin has been shown to be essential for virulence and emergence of FLC tolerance [24], [25]. Inactivation of the pathway by CsA or FK506, or by deletion of the gene encoding one of the calcineurin subunits, in combination with FLC resulted in the killing of cells by this otherwise fungistatic drug [18], [28], [29]. Amiodarone (a calcium channels inhibitor) was also reported to be significantly synergistic with FLC against fluconazole-resistant C. albicans in a calcineurin/Crz1p dependent manner [30], [31]. Several lines of evidence suggested that iron, *AGE3* and *HSP90* mediated antifungal drug susceptibilities cross talk with the calcineurin signaling pathway [17], [32], [33], [34] and calcineurin could be targeted to enhance FLC therapy [16], [35], [36]. Our previous study showed that the disruption of *RTA2* could block the emergence of calcium-mediated tolerance to FLC by aggravating its impairment to the plasma membrane of *C. albicans* [22]. The expression of *RTA2* was found to be under the control of the Ca^2+^-activated-calcineurin *via* its transcriptional factor Crz1p in *C. albicans* [22], [23].

In the present study, we found that *RTA2* itself was not involved in *C. albicans* virulence (Figure 2). However, the disruption of *RTA2* significantly augmented the therapeutic efficacy of FLC against systemic candidiasis. Conversely, both re-introduction of one *RTA2* allele and ectopic expression of *RTA2* significantly reduced FLC efficacy in a murine model of systemic candidiasis (Figure 2 and 3). Furthermore, calcium-activated-calcineurin could not complement the *in vitro* calcium-mediated tolerance to FLC and attenuate its impairment to the plasma membrane of *C. albicans* when genetically compromising the function of Rta2p (Table 2 and Figure 1). The consistent conclusion was drawn by the *in vivo* experiments that FLC treatment was significantly more efficacious when *CRZ1* was restored in *crz1*Δ/Δ *rta2*Δ/Δ mutant but not *crz1*Δ/Δ mutant (Figure 4). Taken together, our study implies that calcium-activated-calcineurin mediates the *in vitro* and *in vivo* responses of *C. albicans* to FLC *via* Rta2p, which is an important determinant of FLC tolerance in *C. albicans*. Given the critical roles by Rta2p in controlling the efficacy of FLC, Rta2p can be an attractive target for the development of powerful and effective therapy strategies against life-threatening fungal infections. However, the molecular mechanisms involved remain unknown. Our previous studies showed that Rta2p acted as a sphingolipid long chain base transporter protein and was critical for the association of certain transmembrane (TM) and glycosylphosphatidylinositol (GPI)-anchored proteins with lipid rafts in *C. albicans* [22], [37]. And ceramide biosynthesis is also essential for the association of TM and GPI-anchored proteins with lipid rafts [38], [39], [40]. In *Saccharomyces cerevisiae*, calcineurin signaling is necessary for normal sphingolipid metabolism by regulating ceramide production [41], [42]. Therefore, we speculate that Rta2p, as a target of the calcineurin pathway, may mediate *in vitro* and *in vivo* responses of *C. albicans* to FLC by regulating the biosynthesis of ceramides.

During an infection, the *C. albicans* cells exist in the host body and are surrounded by blood and other body fluid, where they encounter the antifungal drugs. Previous studies have demonstrated that biological fluids such as serum can have profound effects on antifungal susceptibilities [43], [44], [45], [46]. Of particular note was the blood ionized calcium (0.94–1.33 mM) [26], which was *in vitro* sufficient to activate the protein phosphatase activity of calcineurin in *S. cerevisiae* and *C. albicans* [22], [47], [48]. However, the standard antifungal susceptibility testing [49], which is conducted in RPMI-1640 media containing only 0.42 mM ionized calcium [50], is unreliable in predicting the clinical outcome of therapies, especially for systemic infections [51], [52]. Our finding may partially explain the existence and persistence of such a discrepancy between the susceptibilities of *in vivo* and *in vitro* environments.

## Materials and Methods

### Drugs and Agents

FLC was from Pfizer Inc (New York, N.Y.). Caspofungin was purchased from Merck (Whitehouse Station, N.J.). RPMI 1640 medium (with L-glutamine and without sodium bicarbonate), morpholinepropanesulfonic acid (MOPS), and CaCl_2_ were purchased from Sigma Chemical Co. (Cleveland, Ohio).

### *C. albicans* Strains and Culture Media

*C. albicans* strains used in this study are listed in Table 1 and cultured in YPD medium (1% yeast extract, 2% Bacto peptone, and 2% dextrose) or SC medium (0.67% yeast nitrogen base with ammonium sulfate without amino acids, 2% glucose, 0.077% complete supplement mixture minus uracil) supplemented with 50 µg/mluridine as required.

### Construction of Mutant Strains

All the primer sequences are listed in Table S1. The *XhoI* digested fragment of pUC-RTA2-URA3, constructed by us previously [22], was transformed into the *ura3*Δ/Δ *cna*Δ/Δ (DSY2101) or *ura3*Δ/Δ *crz1*Δ/Δ (MKY59) mutant (Table 1) by standard methods [53].

### Construct of the CNA and CRZ1 Revertant Strains

The genes (*CNA, CRZ1* and *RTA2*) containing their ORFs and 1 kb up/downstream were amplified by PCR with Pyrobest polymerase (TaKaRa). The *PstI-KpnI* digested PCR fragments of *CNA* and *RTA2* was ligated into plasmid pBes116 [54], and then plasmids pBes-CNA and pBes-RTA2 were obtained.The *NotI*-*KpnI* digested PCR fragment of *CRZ1* was ligated into pBes116, and plasmid pBes-CRZ1 was obtained. And then, the AscI digested fragment of pBes-CNA, pBes-CRZ1 or pBes-RTA2 was transformed into DJY201 (*cna*Δ/Δ *rta2*Δ/Δ), MJY201 (*crz1*Δ/Δ *rta2*Δ/Δ) or JXM101 (*rta2*Δ/Δ) as before [22].

### Construct of RTA2 Fused to pCDR2 in *RTA2* Mutants

The fragment containing *CDR2* promoter sequence and ORF of *RTA2* was obtained by the Fusion PCR methods. The *PstI*-*KpnI* digested PCR fragment was ligated into pBes116, and recombinant plasmid pBesCDR2-RTA2 was obtained. DNA sequencing confirmed that the sequence of the insert was identical to *CDR2* and *RTA2* sequence reported in the *Candida* Genome Database (http://www.candidagenome.org/). The *C. albicans* mutant DSJ101 with deletion of drug-resistance related genes including *CDR1*, *CDR2*, *CaMDR1*, *FLU1* and *RTA2* was transformed with the linearized plasmid pBesCDR2-RTA2 by *AscI* and selected on SC medium without uridine as before [22].

### Relative Quantification of Genes by Quantitative RT-PCR

All the primer sequences are listed in Table S1. Quantitative RT-PCR was done as described previously [22].

### Susceptibility Testing

Drug sensitivities were assayed by the modified broth microdilution method as described previously [22]. Briefly, RPMI 1640 medium was adjusted to pH 7 at 25°C using 3-[N-morpholino]propanesulphonic acid (MOPS).The initial concentration of the fungal suspension in the RPMI 1640 medium was 1–5×10^3^ c.f.u./mL, 0.1 ml of cells were inoculated into successive wells of a 96-well microtiter plate containing serial two-fold dilutions of antifungal drugs. Drug-free medium with fungi and a fungi-free medium were used as the positive and negative controls, respectively. After incubation at 35°C for 48 h, absorbance at 630 nm was determined in a microplate reader. MIC_80_ were estimated. Assays for susceptibility to FLC and caspofungin were also performed in the presence of a fixed concentration (1 mM) of CaCl_2._

### Transmission Electron Microscopy

With or without treatment of FLC, CaCl_2_ or FLC plus CaCl_2_, transmission electron microscopies of *C. albicans* cells including the *crz1*Δ/Δ::*CRZ1*, *rta2*Δ/Δ *crz1*Δ/Δ::*CRZ1* and *crz1*Δ/Δ *rta2*Δ/Δ::*RTA2* complemented strains (Table 1) were obtained according to our previously described protocol [22].

### Systemic Murine Candidiasis Model

Groups of ICR female mice (18–22 g) were inoculated *via* lateral tail vein with 200 µl of a suspension containing 1×106 c.f.u different *C. albicans* strains in sterile saline. The following regimens were administered to the infected mice: FLC at 1 mg/kg or 2 mg/kg once a day intraperitoneally for a week, or placebo once a day. Mice were monitored daily for survival for a period of 30 days. Duplicate independent experiments were conducted (n = 10 in each group). Kaplan–Meier and Life Table analyses were used to estimate survival probabilities. Log-rank testing was used to evaluate the equality of survival curves. *P*<0.05 was considered significant.

### Kidney c.f.u. Assay and Histopathological Analysis

Paired kidneys of untreated and FLC-treated mice were removed aseptically on day 3 and 8 post inoculation, which were weighed and homogenized in 5 ml sterile physiological saline. Serial dilutions were carried out in Sabouraud chloramphenicol agar to determine the c.f.u./g kidney. Statistical analyses were performed using anova and post hoc (Bonferroni and Student–Newman–Keuls’) tests. *P*<0.05 was considered significant. For histopathological analysis, paired kidneys of untreated and FLC-treated mice were removed aseptically on day 3 and 8 post infection from each mouse before being fixed in 10% neutral buffered formalin. Kidneys were stained using haematoxylin and eosin (H&E) and periodic acid-Schiff (PAS) to reveal inflammatory infiltration, microabscesses, abscesses and necrosis (H&E staining) and the hyphal structure of the fungal pathogens (PAS). Tissues were examined microscopically.

### Ethics Statement

The animal experiments were approved by the Animal Ethics Committee of Second Military Medical University (Shanghai, China). All surgery was performed under sodium pentobarbital anesthesia, and all efforts were made to minimize suffering of the animals.

## Supporting Information

Figure S1 **Schematic representation of disruption of** ***RTA2*** **using the** ***URA3*** **selection marker. (A)** Sequential targeted disruption of the two *RTA2* alleles in *C. albicans* with the disruption cassette. **(B)** Southern analysis of the genomic DNA digested with BglII and SalI. The exact size and genotype of the expected hybridizing DNA fragment are indicated on the right.
(TIF)

Figure S2 **(A)** Strains, with one allele of *RTA2* reintroduced into *ADE2* locus, yielded only one 3.8 kb PCR product by PCR analysis with primer specific to *ADE2* and primer specific to *RTA2*, with plasmid pBes-RTA2 as control. **(B)** Strains, with one allele of *CNA or CRZ1* reintroduced into *ADE2* locus, yielded only one 2.5 kb PCR product by PCR analysis with primer specific to *ADE2* and primer specific to *URA3*, with plasmid pBes116 as control. **(C)** Strains, with the fusion fragment of *CDR2* promoter and ORF of *RTA2* reintroduced into *ADE2* locus, yielded only one 3.9 kb PCR product by PCR analysis with primer specific to *ADE2* and primer specific to *RTA2*, with plasmid pBesCDR2-RTA2 as control.
(TIF)

Figure S3 **(A)** Expression levels of *UTR2* were examined by quantitative RT-PCR in the wild-type strain (CAF2-1), DJYCNA (*rta2Δ/Δ cnaΔ/Δ*::*CNA*) and MJYCRZ1 (*rta2Δ/Δ crz1Δ/Δ*::*CRZ1*) after exposure to CaCl_2_ (200 mM) for 2 h, with their corresponding drug-free strains as controls. **(B)** Expression levels of *RTA2* were examined by quantitative RT-PCR in the wild-type strain (CAF2-1), DJYRTA2 (*cnaΔ/Δ rta2Δ/Δ*::*RTA2*) and MJYRTA2 (*crz1Δ/Δ rta2Δ/Δ*::*RTA2*) after exposure to CaCl_2_ (1 mM) for 16 h, with their corresponding drug-free strains as controls. **(C)** Expression levels of *RTA2* were examined by quantitative RT-PCR in the parental strain (DSJ101U), DSJ-RTA2 carrying the fusion of the oestradiol-inducible *CDR2* promoter and ORF of *RTA2,* in the presence of oestradiol (OST, 10 ug/ml), with their corresponding drug-free strains as controls. Data are represented as means ± standard deviation.
(TIF)

Figure S4 **Schematic view of the calcineurin pathway in** ***Candida albicans.***
(TIF)

Table S1 **Primers used in this study.**
(DOCX)
