# Correction: Calcium-Activated-Calcineurin Reduces the *In Vitro* and *In Vivo* Sensitivity of Fluconazole to *Candida albicans via* Rta2p

**DOI:** 10.1371/journal.pone.0317176

**Published:** 2025-01-03

**Authors:** Yu Jia, Ren-Jie Tang, Lin Wang, Xiang Zhang, Ying Wang, Xin-Ming Jia, Yuan-Ying Jiang

In Fig 1, the panel C is uploaded incorrectly. Please see the correct Fig 1 here.

**Fig 1:**
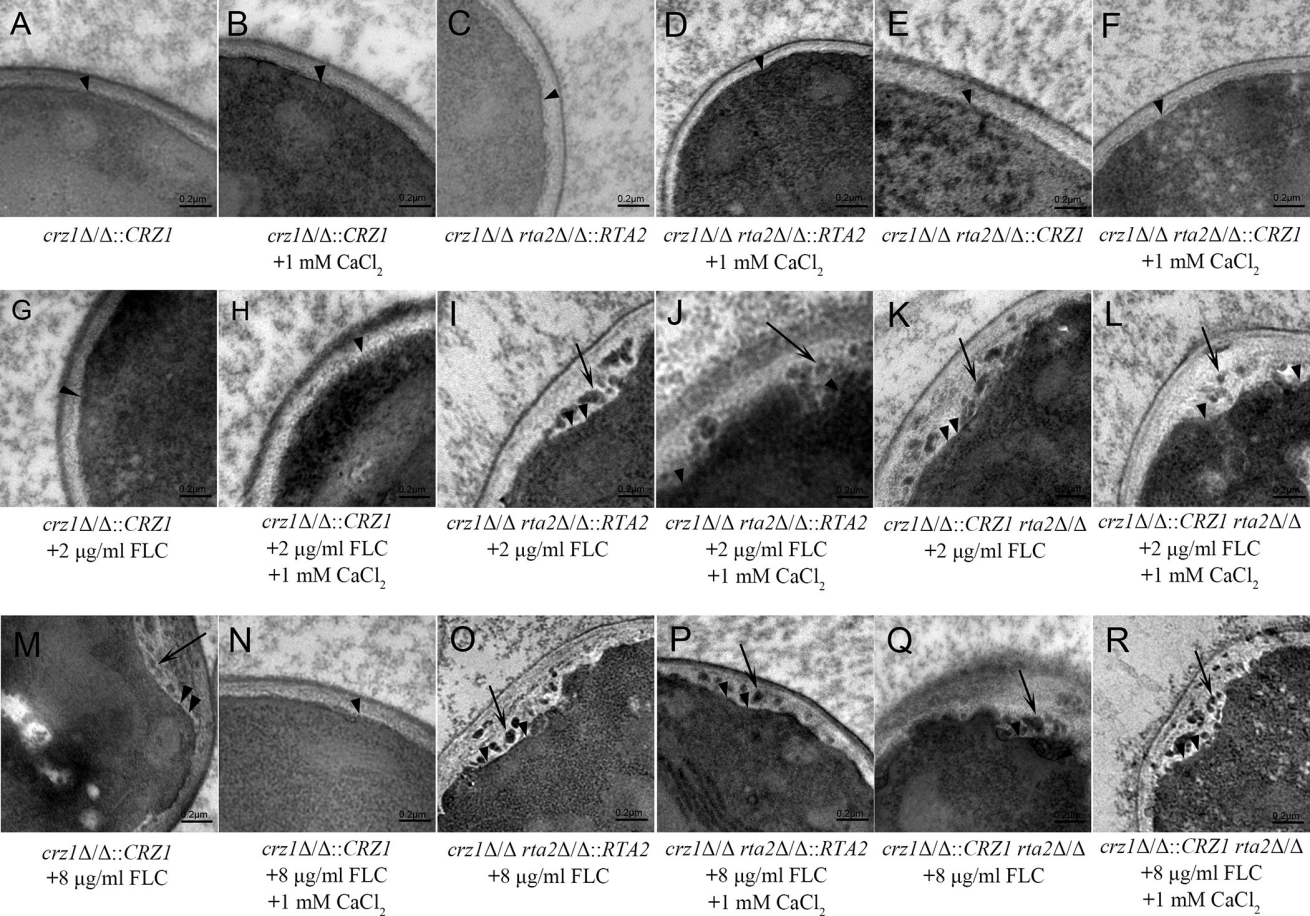
Ultra-structure of *C*. *albicans* cells. (**A to R**) Ultra-structural images of *crz1*Δ/Δ::*CRZ1*, *rta2*Δ/Δ *crz1*Δ/Δ::*CRZ1* and *crz1*Δ/Δ *rta2*Δ/Δ::*RTA2* complemented *C*. *albicans* strains in the presence or absence of agents at indicated concentrations shown on the lower. Arrow heads indicated the cytoplasmic membrane and arrows indicated the extensive solubilization of the cytoplasmic membrane. The bar represents a length of 0.2 μm.
