# Supplementary figures and images for: Calcium-Activated-Calcineurin Reduces the In Vitro and In Vivo Sensitivity of Fluconazole to Candida albicans via Rta2p
Source: PLoS One. 2012 Oct 30;7(10):e48369. doi: 10.1371/journal.pone.0048369 (PMC3484117; doi:10.1371/journal.pone.0048369)

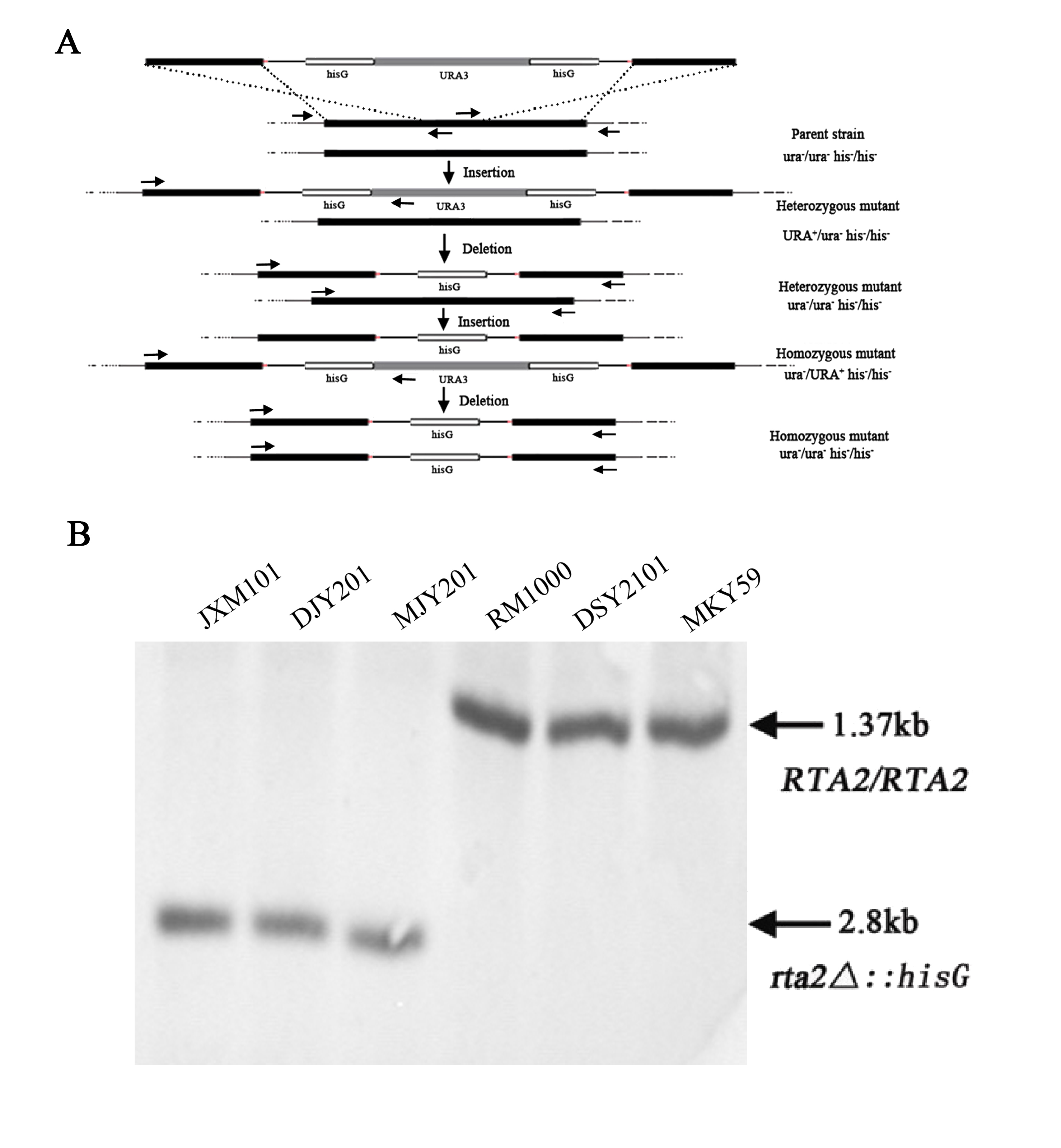

Supplement: Figure S1 — Schematic representation of disruption of RTA2 using the URA3 selection marker. (A) Sequential targeted disruption of the two RTA2 alleles in C. albicans with the disruption cassette. (B) Southern analysis of the genomic DNA digested with BglII and SalI. The exact size and genotype of the expected hybridizing DNA fragment are indicated on the right. (TIF) [file pone.0048369.s001.tif]

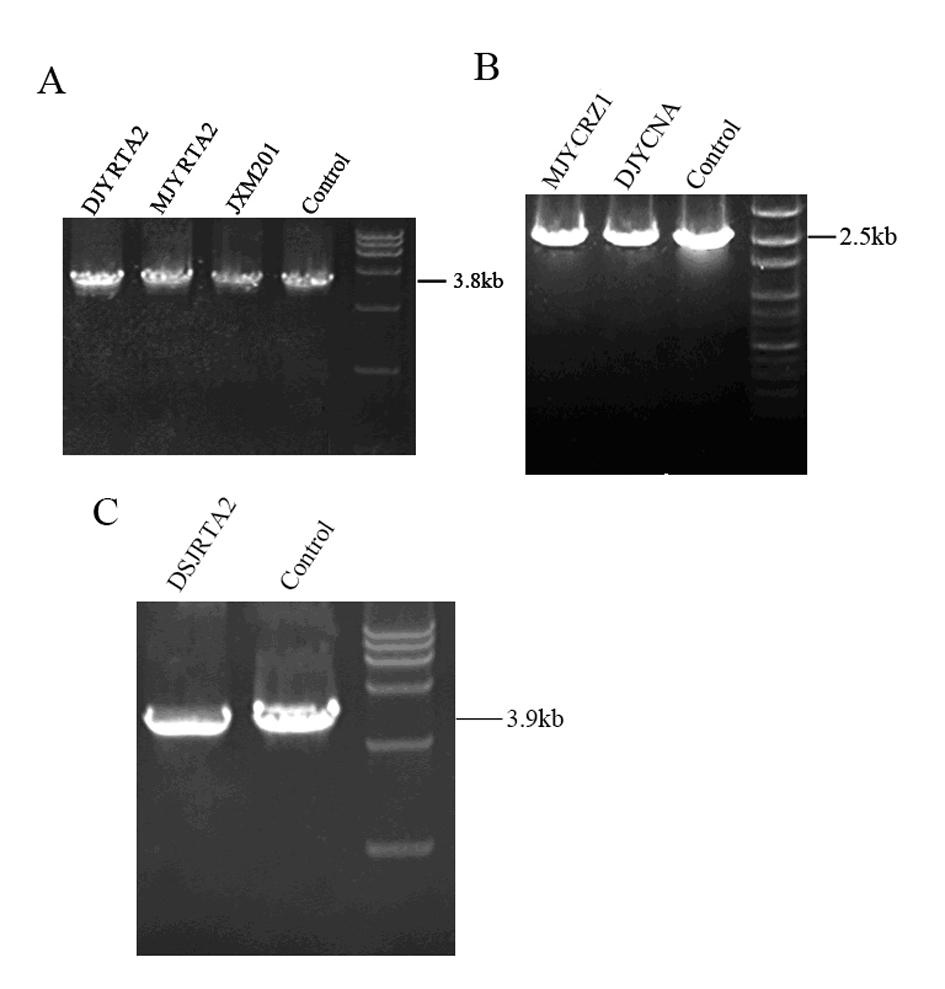

Supplement: Figure S2 — (A) Strains, with one allele of RTA2 reintroduced into ADE2 locus, yielded only one 3.8 kb PCR product by PCR analysis with primer specific to ADE2 and primer specific to RTA2, with plasmid pBes-RTA2 as control. (B) Strains, with one allele of CNA or CRZ1 reintroduced into ADE2 locus, yielded only one 2.5 kb PCR product by PCR analysis with primer specific to ADE2 and primer specific to URA3, with plasmid pBes116 as control. (C) Strains, with the fusion fragment of CDR2 promoter and ORF of RTA2 reintroduced into ADE2 locus, yielded only one 3.9 kb PCR product by PCR analysis with primer specific to ADE2 and primer specific to RTA2, with plasmid pBesCDR2-RTA2 as control. (TIF) [file pone.0048369.s002.tif]

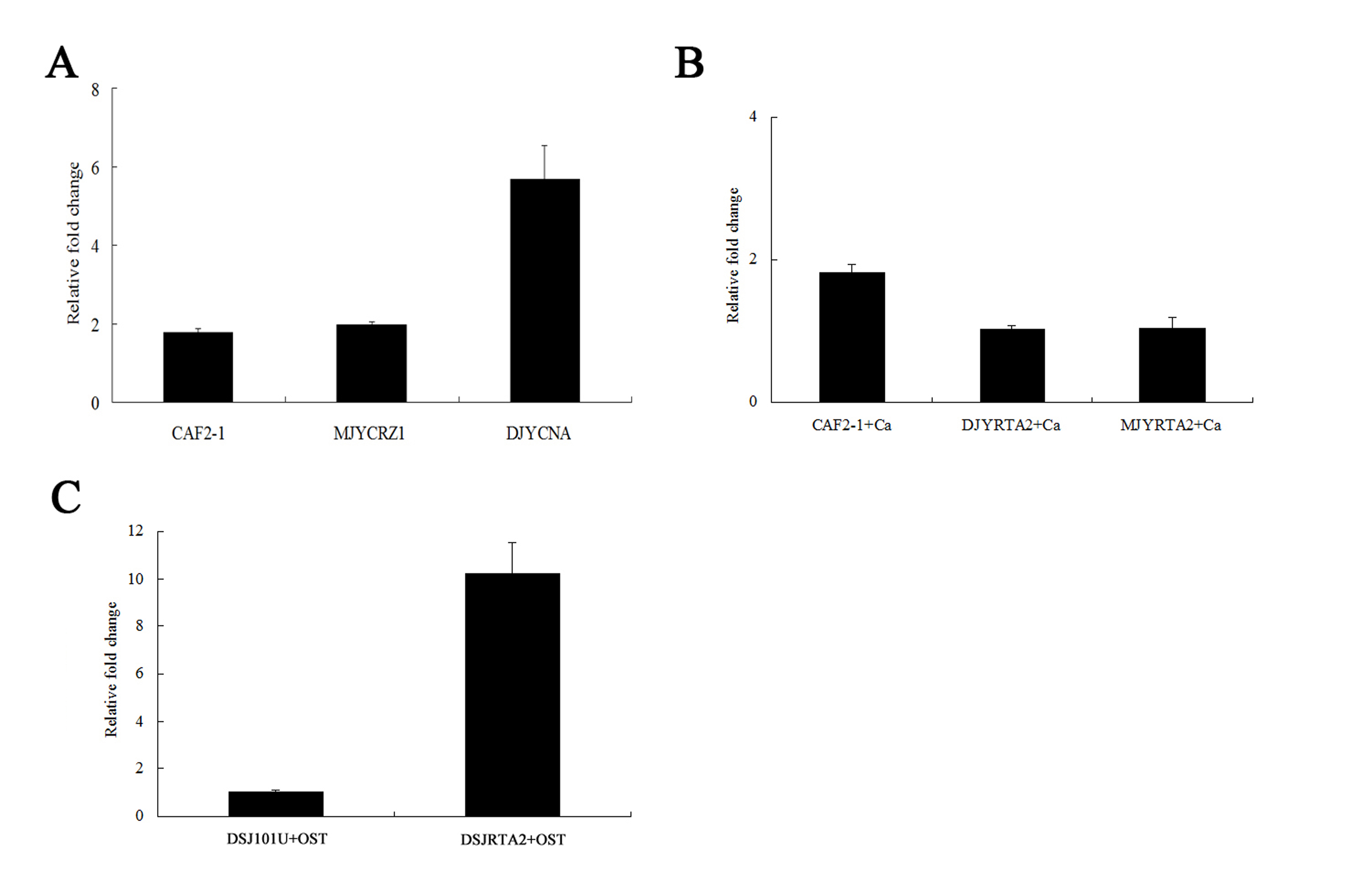

Supplement: Figure S3 — (A) Expression levels of UTR2 were examined by quantitative RT-PCR in the wild-type strain (CAF2-1), DJYCNA (rta2Δ/Δ cnaΔ/Δ::CNA) and MJYCRZ1 (rta2Δ/Δ crz1Δ/Δ::CRZ1) after exposure to CaCl2 (200 mM) for 2 h, with their corresponding drug-free strains as controls. (B) Expression levels of RTA2 were examined by quantitative RT-PCR in the wild-type strain (CAF2-1), DJYRTA2 (cnaΔ/Δ rta2Δ/Δ::RTA2) and MJYRTA2 (crz1Δ/Δ rta2Δ/Δ::RTA2) after exposure to CaCl2 (1 mM) for 16 h, with their corresponding drug-free strains as controls. (C) Expression levels of RTA2 were examined by quantitative RT-PCR in the parental strain (DSJ101U), DSJ-RTA2 carrying the fusion of the oestradiol-inducible CDR2 promoter and ORF of RTA2, in the presence of oestradiol (OST, 10 ug/ml), with their corresponding drug-free strains as controls. Data are represented as means ± standard deviation. (TIF) [file pone.0048369.s003.tif]

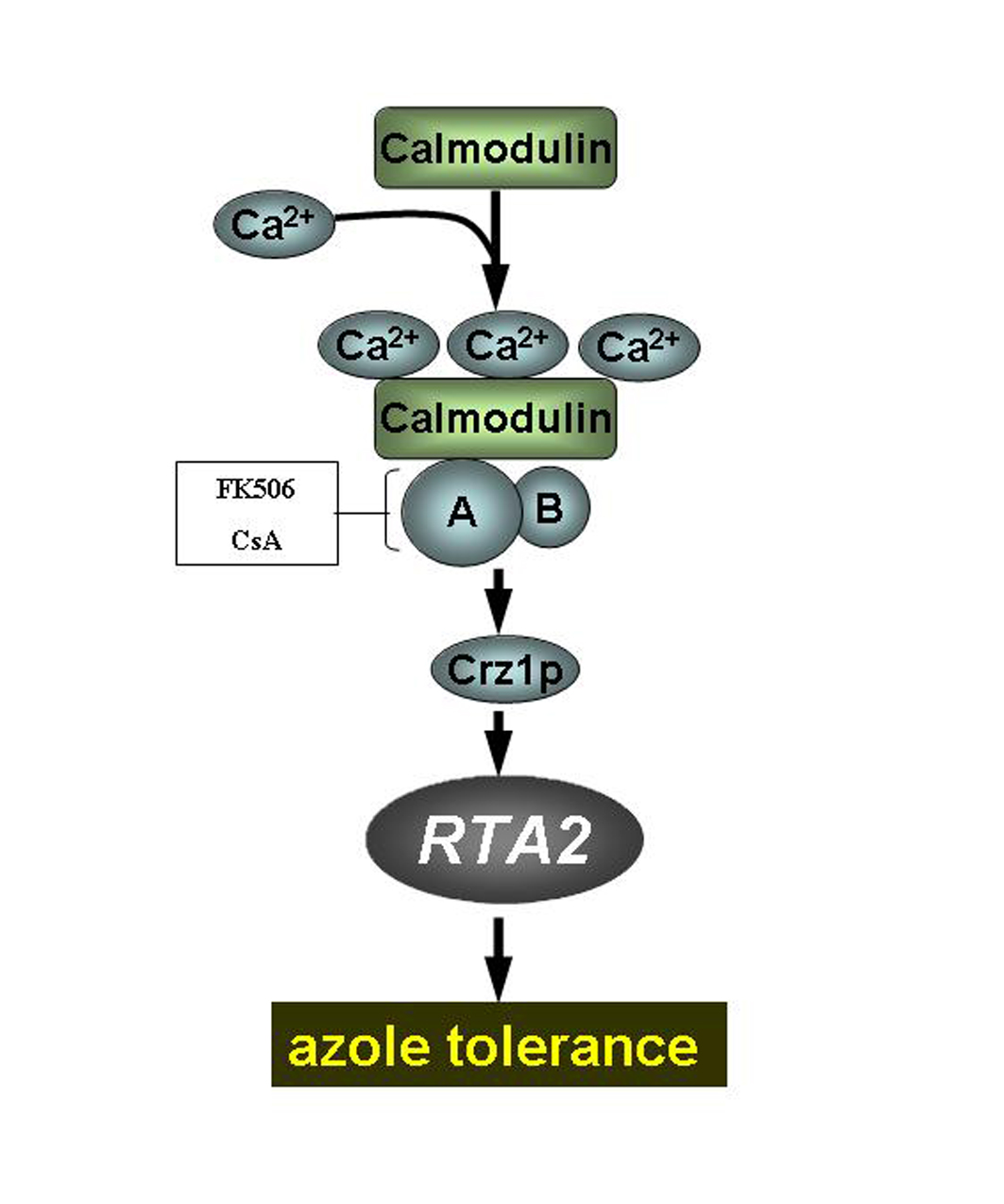

Supplement: Figure S4 — Schematic view of the calcineurin pathway in Candida albicans. (TIF) [file pone.0048369.s004.tif]
