# Supplementary material for: Calcium-Activated-Calcineurin Reduces the In Vitro and In Vivo Sensitivity of Fluconazole to Candida albicans via Rta2p
Source: PLoS One. 2012 Oct 30;7(10):e48369. doi: 10.1371/journal.pone.0048369 (PMC3484117; doi:10.1371/journal.pone.0048369)
Supplement: Table S1 — Primers used in this study. (DOCX) [file pone.0048369.s005.docx]

**Table S1 Primers used in this study**

| Primer | sequence |
| --- | --- |
| **Primers for construction of the *RTA2,CNA or CRZ1* revertant strain** | |
| CNA- FWD | AAAACTGCAGCATCAAACCCCCAAAGTTTATG |
| CNA- RV | CGGGGTACCCCAAAGACGAATCTCGACTG |
| CRZ1- FWD | ATAAGAATGCGGCCGCCGCATTTTTGAAATCCAGCAG |
| CRZ1- RV | CGGGGTACCGATGATGATGATGATGGAACCG |
| **Primers for verifying the integration of intact *RTA2* in the *ADE2* site** | |
| ADE2chk- FWD | TCTCAGTCAACCATTTTGAGTC |
| RTA2chk- RV | GTAGGTGTGGAAGTTGGAGTCC |
| **Primers for verifying the integration of intact *CNA or CRZ1* in the *ADE2* site** | |
| ADE2chk- FWD | TCTCAGTCAACCATTTTGAGTC |
| **Primes for construction of *RTA2* fused to pCDR2 in *rta2* mutants** | |
| CDR2-FWD | AACTGCAGTGCAAAAACTGATAATATACCTCTG |
| CDR2-RV | **ATGTTTTTATTGTATGTGTTAATTAGTGAA** |
| RTA2-FWD | **TTCACTAATTAACACATACAATAAAAACAT**TCCCACCCTTCAACTATGAG |
| RTA2-RV | GGGGTACCTGCGTACCAAAAGTCATCG |
| **Primes for verifying the integration of *CDR2* promoter and *RTA2* ORF fused fragment in the *ADE2* site** | |
| ADE2chk- FWD | TCTCAGTCAACCATTTTGAGTC |
| RTA2chk-RV | CATTAAATGACAACACTCGTCC |
| URA3chk- RV | CAGTGCTAACAACTTCATCAAC |

The underlined sequence corresponds to the restriction enzyme site,and the overlap sequence are in bold.
